# Lipid Profiles and Cardiovascular Risk in Patients with Oral Lichen Planus

**DOI:** 10.3390/dj10040061

**Published:** 2022-04-06

**Authors:** Tomislav Radic, Livia Cigic, Ana Glavina, Ana Hrboka, Ana Druzijanic, Ivona Musa Leko, Dolores Biocina-Lukenda

**Affiliations:** 1 Dental Polyclinic Arena, Zagreb, Remetinecki gaj 2k, 10000 Zagreb, Croatia; tomo_1992@yahoo.com; 2 Department of Oral Medicine and Periodontology, School of Medicine, University of Split, Soltanska 2, 21000 Split, Croatia; dlukenda@mefst.hr; 3 Department of Dental Medicine, University Hospital of Split, Spinciceva 2, 21000 Split, Croatia; ana.druzijanic8.1.ad@gmail.com; 4 Dental Polyclinic Split, Matoseva 2, 21000 Split, Croatia; aglavina@mefst.hr; 5 Private Dental Practice Ana Hrboka, Kralja Kresimira 1/1, 21214 Kastel Kambelovac, Croatia; anahrboka@gmail.com; 6 Dental Polyclinic Musa, Kralja Tomislava 78, 88000 Mostar, Bosnia and Herzegovina; ivonamusaleko@gmail.com

**Keywords:** oral lichen planus, lipid profile, Castelli index, cardiovascular risk

## Abstract

Background: It has been reported that dyslipidemia prevalence and cardiovascular disease risk were increased in subjects with oral lichen planus diagnose. On the other hand, so far, there is no available data on the topic of cardiovascular risk (CVR) in subjects with oral lichen planus (OLP). The main aim of this study, due to lack of any other study covering this topic, was to investigate lipid profile and assess CVR in patients with OLP. Materials and Methods: To create a routine lipid profile, we collected triglyceride serum levels, total cholesterol, low-density lipoprotein cholesterol, and high-density lipoprotein cholesterol from 63 OLP patients and 63 healthy people representing control subjects. For every patient their individual cardiovascular risk was measured. Results: In comparison with the tested control subjects, patients with OLP had all parameters of the lipid profile elevated, with no differences of statistical importance. Furthermore, the experimental (OLP) and control groups shared similar mean values of the lipid profile parameters. Conclusions: The association of OLP with cardiovascular risk was not established and further studies with more subjects involved are required to validate this connection.

## 1. Introduction

Oral lichen planus (OLP) is a disease with chronic and inflammatory features that have a pathological effect on the membrane of oral mucosa. OLP mainly affects patients of middle age or older and is more common in female patients. The aetiology of lichen planus still remains unknown. In contrast, the pathogenesis of this chronic inflammatory disease is relatively well understood. It is known that OLP creates immunoreaction directed against the basal cells situated in the oral epithelium [1]. The basal epithelial cells die by apoptosis. It is unclear whether this process is driven by an antigen-specific mechanism or whether it is reaction to an extrinsic antigen. OLP is a very common chronic disease with a prevalence of 1.27–2.0% in the general population, according to existing data [2]. The oral lesions have a characteristic appearance and distribution. Lesions are usually bilateral and symmetrical. The most common OLP clinical forms are papular, atrophic, plaque-like, erosive, reticular, and bullous. Furthermore, OLP is mostly located on the mucosa of the buccal surface, dorsum of the tongue and gingiva. Any one patient may have several presentations that change from one pattern to another over time. OLP patients may feel pain or a burning sensation but can also be asymptomatic. Asymptomatic lesions usually require no treatment. Oral lesions in erosive forms of OLP often cause pain or burning sensations and require treatment. It is usual to start therapy with low-potency topical steroids. Systemic corticosteroids are reserved for the short-term treatment of severe cases of the disease where topical approaches have failed. The diagnosis of OLP can usually be made on the history, the appearance, and the distribution of the lesions. Biopsy of oral white and plaque-type lesions is required to exclude dysplasia [1,2,3]. This is of special importance due to OLP potential malignant transformation. The risk of malignant change in OLP lesions has long been controversial, and specific risk factors have not been identified [3]. OLP can also be associated with other comorbidities such as diabetes mellitus, metabolic syndrome, dyslipidemia, thyroid disease, psychosomatic ailments, gastrointestinal disease, chronic active hepatitis, and bowel diseases [2,3].

Lipid raft is a term which is mostly used to give a precise description of certain microdomains found in the plasma membrane. Thanks to their lipid composition, those microdomains are in a liquid-ordered phase. They are structurally full of glycosphingolipids, sphingomyelin, and cholesterol [4].

Cholesterol, an essential element for the normal function of all human cells, has various other biological functions. Cholesterol composes about 30% of all animal cell membranes, regulates the biological process of substrate presentation, and is implicated in cell signaling processes. A lipid profile is a panel of blood tests used to find abnormalities in lipids, such as cholesterol and triglycerides. The results of this test can identify certain genetic diseases and can determine approximate risks for cardiovascular disease [4,5]. It has been reported in several studies that lipid rafts have the most important impact on signalling receptor and activation of lymphocytes, especially the early phase of stimulation T cell receptors. Both, cholesterol and triglyceride in high levels are major causes of hyperlipidaemia [5]. Furthermore, malignancy has been associated with changes in lipid profile because of the key role that lipids play in maintaining the integrity of the cell membrane. Changes in plasma lipid profile have been observed in patients with cancers of various organs [6]. It is well-known that oral lichen planus is recognized as an oral *potentially malignant disorder* with a higher potential for malignant alteration than normal oral mucosa. A number of patients develop carcinoma in a background of keratosis and atrophy, although the overall transformation rate in oral lichen planus seems unlikely to exceed 0.05% in ten years [3]. Therefore, the logical question is whether there is a change in the lipid profile in patients with oral potentially malignant disorder, such as oral lichen planus.

Prolonged dyslipidaemia caused by chronic inflammatory conditions supports the formation of atherosclerotic plaques that are associated with an increased CVR [7]. Some of the well-known autoimmune skin diseases, such as alopecia and psoriasis, are related to abnormalities in lipid structure and increased risk for cardiovascular disease [8]. There is also evidence of an association between lichen planus and significant dyslipidaemia [9]. Another case-control study has also shown that patients with lichen planus, particularly those with existing effect on mucosa, have a higher incidence of metabolic syndrome, which is linked with a risk of cardiovascular diseases [10]. Some studies showed that OLP patients have a more damaged lipid metabolism than patients with skin lichen planus or the general population [11,12,13]. However, there is not enough evidence to establish the cardiovascular risk in patients with OLP.

Investigation and comparison of lipid profiles and cardiovascular risk in subjects with OLP diagnosis and the healthy control group were the main aims of this study.

## 2. Material and Methods

### 2.1. Participants

The Ethics Committee of the University of Split, School of Medicine gave consent for the study. It was conducted according to the principles of the Declaration of Helsinki. Signed informed agreement was filled by all study participants before the beginning of the study. Sixty-three participants diagnosed with OLP from the Dental Polyclinic Split at the University of Split, School of Medicine were prospectively enrolled in the study between October 2012 and October 2016. All participants took part in extensive interviews and large clinical examinations. It was all conducted by the same oral medicine specialist. OLP diagnosis was confirmed in all subjects through pathohistological diagnosis. After administrating 1 mL of local anaesthetic solution biopsy samples were taken from the edge of the pathological oral mucosal membrane. Furthermore, OLP can be diagnosed after satisfying specific histological criteria that include the following: hyperkeratosis and variable degrees of orthokeratosis or parakeratosis, vacuolization with apoptotic keratinocytes in the basal layer, lymphophagocytic infiltrate found on the epithelium–connective tissue interface, the existence of eosinophilic colloid bodies at the basal epithelium (Civatte bodies), and sawtooth-shaped interpapillary ridges. The histological detection in cases clinically accordant with erosive forms also included epithelial erosion, as well as neutrophils and fibrin deposits in the epithelium [3]. Thirteen patients had a non-erosive form of the disease based on the clinical and pathohistological findings, while 50 patients had an erosive form of OLP.

While collecting a medical history, participants were asked about their age, smoking status, and alcohol consumption. Data on alcohol consumption were obtained during the anamnesis, and were categorized as “yes” or “no”. The cut-off for “yes” averaged more than two drinks per day for seven days in the last month. Measurements of height and weight were obtained by using a stadiometer and a balance beam scale at the first visit in order to calculate body mass index (BMI) for each patient involved in the study. Body mass index was mathematically expressed as kilograms per square metre. Subjects were also asked about the existence of hypertension. Blood pressure (BP) measurements were performed in triplicate for each subject while they were sitting followed by 10 min of resting at 1 min intervals. A mercury sphygmomanometer was used to properly calculate blood pressure. High blood pressure was determined as BP ≥ 140/90 mm Hg on several occasions.

All participants with a previous diagnosis of autoimmune disease and participants already involved in any lipid-lowering therapy were excluded from this study.

The control group subjects (*N* = 63) were also found in the Dental Polyclinic Split at the University of Split, School of Medicine from the base of healthy patients. Their clinical check-up and epithelium evaluation showed no signs of any oral lesions. Patients in the control group did not have any chronic disease in their medical history, nor did they take any medications.

### 2.2. Laboratory Analysis

Blood samples of fasting subjects were provided from the antecubital vein. Samples were taken early in the morning (7 a.m.–8 a.m.) and they consisted of total cholesterol (TC), low-density lipoprotein cholesterol (LDL-c), high-density lipoprotein cholesterol (HDL-c), and triglycerides (TG) serum levels. All of the parameters were acquired to create an individual lipid profile for every subject involved in the study. The collected samples were all analysed following standard protocols by the same experienced biochemist and in the same laboratory.

#### 2.2.1. Castelli Index and Cardiovascular Risk

Castelli index 1 (TC/HDL-c) and Castelli index 2 (LDL-c/HDL-c) were calculated. The average ratio of Castelli index 1 in healthy individuals is approximately 3.5 or lower, and for Castelli index 2, it is 3 or lower.

Cardiovascular risk was also calculated for every involved subject according to the parameters of the European society of cardiology low cardiovascular risk chart. Existing parameters were presented as age, gender, total cholesterol, systolic blood pressure, and smoking status, as recommended by cardiologists [14].

#### 2.2.2. Statistical Analysis

STATISTICA 11.0 software package was used for statistical evaluation. Principal statistical parameters were calculated by a fundamental statistics method and frequency tables. The difference between the control and experimental groups for continuous variables was assessed by Student’s t-test and for categorical variables by the Mann–Whitney U Test. The difference between categories was estimated by an χ^2^ test. The influence of the predictor variables on the presence/absence of OLP was tested by multiple regression analysis and a general regression model. *p* < 0.05 was the importance level in all tests.

## 3. Results

### 3.1. Participant Baseline Characteristics

The experimental group contained 63 patients who had confirmed clinical and histopathological diagnoses of OLP. On the other hand, the involved control group of 63 healthy subjects had no oral lesions. There were 9 males (14.3%) and 54 females (85.7%) in the experimental group and 14 males (22.2%) and 49 females (77.8%) in the control group. No statistically important gender differences were observed between these groups (*p* = 0.25). Basic statistical parameters for age, body mass index, cholesterol and triglyceride levels, calculated Castelli indexes, and calculated cardiovascular risk for the experimental and control groups are indicated in Table 1.

When comparing percentage and the number of the people in the experimental and control groups with cholesterol values and triglycerides that exceeded the normal range, a higher incidence was obtained in the experimental group (Table 2).

There was no difference in lipid profile or cardiovascular risk between the clinical form of oral lichen planus (erosive vs. non-erosive form). Statistically, no important difference could be found between the groups in our study for high blood pressure, which was detected in 26 (41.3%) OLP and 24 (38.1%) control subjects. A lower percentage of smokers (11.1%) was found in the experimental group in comparison with the control group (17.5%), while it was exactly the opposite (17.5% in the experimental group vs. 11.1% in the control group) for alcohol consumption.

### 3.2. Predictors of the OLP Diagnosis

**Figure 1:**
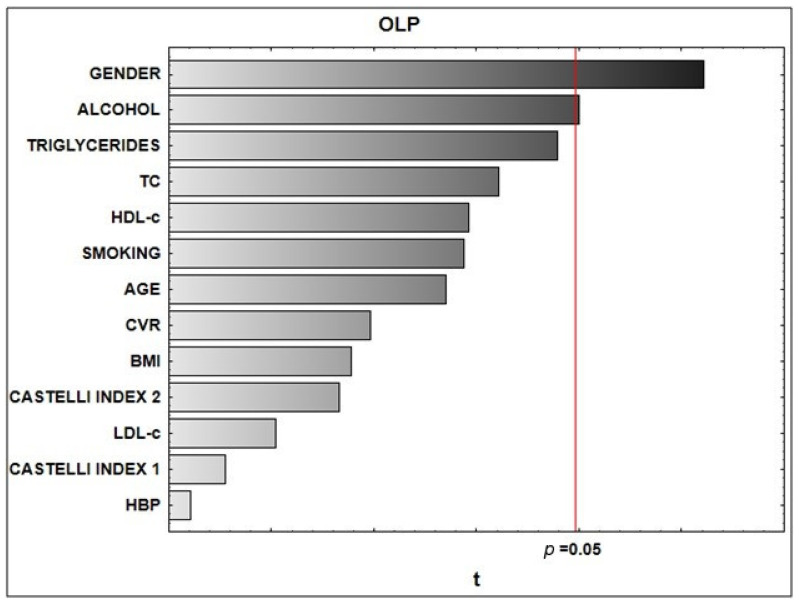
Correlations among the existence/absence of oral lichen planus (diagnosis) and chosen predictor variables.

The link between the presence/absence of OLP (diagnosis) and the chosen predictor variables were introduced through the form of a Pareto chart (Figure 1).

HDL-c, high-density lipoprotein cholesterol; Castelli index 1, calculated as the ratio of total cholesterol/HDL-c; TC, total cholesterol; LDL-c, low-density lipoprotein cholesterol; CVR, cardiovascular risk; body mass index; BMI HBP, high blood pressure; Castelli index 2, calculated as the ratio of LDL-c/HDL-c.

The presence/absence of OLP showed a statistically significant correlation with the chosen variables as predictor ones (R = 0.53; *p* < 0.04). According to beta coefficient values and their significance levels, the statistically significant factors that contributed to the correlation were female gender (β = 0.80; *p* = 0.01) and alcohol consumption (β = 0.75; *p* = 0.02).

## 4. Discussion

This study showed that all tested parameters of lipid profile were more elevated in OLP patients than in the control group but with no statistically significant difference. Chronic inflammation causes disturbances in lipid metabolism. Hyperlipidaemia is the most common form of dyslipidaemia. Ozbagcivan O et al. showed that OLP patients have a more damaged lipid metabolism compared with patients with skin lichen planus [11]. This can be explained by the fact that OLP is a chronic disease and, at the same time, has a longer duration compared to lichen planus. It is important for clinicians to recognize coexisting atherogenic dyslipidemia and to implement early preventive measures in oral lichen planus patients [11]. Lopez-Jornet et al. described a 58% prevalence of dyslipidaemia among patients with OLP and statistically significant differences in high-density lipoprotein cholesterol among OLP patients in comparison to the control subjects without oral lichen planus [12]. Krishnamoorthy et al. also found that patients with oral lichen planus had higher levels of cholesterol in serum and low-density lipoprotein cholesterol in comparison with the general population [13]. In contrast, an additional study by Baykal et al. revealed no important differences between lichen planus patients and controls with respect to the prevalence of dyslipidaemia [10].

In our study, the mean values of total cholesterol were the same in the experimental and control groups. Low-density lipoprotein cholesterol and triglycerides were higher among the experimental patients than they were among the control subjects, whereas high-density lipoprotein cholesterol was lower in the experimental patients. However, no important statistical difference between the OLP group in comparison to the control group was found. Furthermore, there were elevated levels of all lipid profile parameters in more OLP patients compared with the control group. This indicates a possible association between OLP and dyslipidemia and future studies with more subjects involved are crucial to verify this connection. Conditions with chronic and inflammatory manifestation, such as OLP, can cause prolonged dyslipidaemia and increase cardiovascular risk [13]. Lopez-Jornet et al. found a higher Castelli index among OLP patients in their study [12]. Castelli index 1 and Castelli index 2 were calculated [15], and it was found that the mean values tended to be slightly higher in the population with diagnosed OLP in comparison with the control subjects of our study group. However, statistics did not show important differences between the two study groups. We found an increased Castelli index 1 in 34 (53.9%) OLP patients and 31 (49.2%) controls as well as an increased Castelli index 2 in 12 (19.0%) OLP patients and 7 (11.1%) control subjects.

Cardiovascular risk was measured for every involved subject according to the European Society of Cardiology low cardiovascular risk chart. Existing parameters were focused on age, gender, total cholesterol, systolic blood pressure, and smoking habits. The mean values of age were similar in both groups (62 years), and female patients prevailed (85.7% in OLP group), which correlates with data showing that OLP is often more diagnosed in middle-aged and older women [16]. No significant difference was noticed statistically between the study groups for high blood pressure, which was found in 26 (41.3%) OLP and 24 (38.1%) control subjects. Additionally, no differences between the experimental and control groups in smoking status or alcohol consumption were found. A lower percentage of smokers (11.1%) was detected in the experimental group when compared with the control group (17.5%). Furthermore, this can be caused by the fact that patients with OLP are educated that oral lichen is a potentially malignant disorder (PMD) with its possible malignant transformation into oral cancer, and smoking is one of the factors that highly increases the chance of developing it. Smoking is considered a major cardiovascular risk factor. Additionally, there is strong evidence supporting a relationship between body mass index and cardiovascular risk [17]. No difference in body mass index was noted in the OLP group in comparison to the control of our study. The mean values of calculated cardiovascular risk were similar between the experimental group and the control group. Furthermore, no statistically significant differences between oral lichen planus patients and healthy controls were detected. No association was found between OLP and cardiovascular risk during the time of our study.

## 5. Conclusions

We found that all the tested lipid profile parameters had elevated levels in more oral lichen planus patients than control subjects, but with no statistically significant difference. Considering this possible association between lichen planus and dyslipidemia, it is important for clinicians to know that it is indicative to perform a complete blood count and determine lipid profile parameters levels in the diagnostic procedure for patients who have a clinical and histopathological confirmed diagnosis of OLP. We did not find an association between cardiovascular risk and OLP. Given the limitations of the study, further studies with more involved subjects are needed to verify this connection.

## Figures and Tables

**Table 1:** Statistically basic parameters of age, body mass index (BMI), calculated cardiovascular risk, cholesterol and triglycerides level, and calculated Castelli indexes in OLP and control group.

Variable	OLP Group *N* = 63	Control Group *N* = 63	*p*
Age (yrs)	62.62 (9.47)	62.21 (10.75)	0.81
BMI	25.69 (3.66)	26.10 (3.81)	0.54
Cardiovascular risk (%)	3.67 (2.60)	4.08 (2.74)	0.59
Total cholesterol (mmol/L)	5.61 (0.87)	5.61 (0.94)	0.99
HDL-c (mmol/L)	1.56 (0.41)	1.61 (0.43)	0.46
LDL-c (mmol/L)	3.38 (0.94)	3.26 (0.82)	0.42
Castelli index 1	3.80 (1.29)	3.63 (0.89)	0.40
Castelli index 2	2.31 (0.92)	2.14 (0.77)	0.25
Triglycerides (mmol/L)	1.41 (1.06)	1.33 (0.52)	0.59

X presents data, mean value (SD, standard deviation). OLP, oral lichen planus; BMI, body mass index; HDL-c, high-density lipoprotein cholesterol; LDL-c, low-density lipoprotein cholesterol; Castelli index 1 calculated as the ratio of total cholesterol/HDL-c; Castelli index 2 calculated as the ratio of LDL-c/HDL-c.

**Table 2:** Differences in cholesterol and triglyceride values that exceeded normal ranges between subjects with oral lichen planus and the control group.

Variable	OLP Group *N* = 63	Control Group *N* = 63
Total cholesterol	50 (79.4)	43 (68.3)
HDL-c	8 (12.7)	6 (9.5)
LDL-c	42 (66.7)	29 (46)
Triglycerides	13 (20.1)	12 (19)

Data are presented as *N* (%). OLP, oral lichen planus; HDL-c, high-density lipoprotein cholesterol; LDL-c, low-density lipoprotein cholesterol.
